# Regulatory effects of oral microbe on intestinal microbiota and the illness

**DOI:** 10.3389/fcimb.2023.1093967

**Published:** 2023-02-01

**Authors:** Yanbei Lu, Zhengyi Li, Xian Peng

**Affiliations:** State Key Laboratory of Oral Diseases, National Clinical Research Center for Oral Diseases, Chengdu, Sichuan, China

**Keywords:** oral microbe, intestinal microbe, gut diseases, microbiota, immunity, oral-gut axis

## Abstract

Over the past decade, the association between oral health, intestinal microbiota, and systemic diseases has been further validated. Some oral microbial species have been isolated from pathological intestine mucosa or feces and identified as biomarkers for intestinal diseases. A small proportion of oral microbiome passes through or colonizes the lower gastrointestinal tract, even in healthy individuals. Opportunistic pathogens from the oral cavity may expand and participate in the occurrence and progression of intestinal diseases when the anatomical barrier is disrupted. These disruptors interact with the intestinal microbiota, disturbing indigenous microorganisms, and mucosal barriers through direct colonization, blood circulation, or derived metabolite pathways. While interacting with the host’s immune system, oral-derived pathogens stimulate inflammation responses and guide the transition of the intestinal microenvironment from a healthy state to a pre-disease state. Therefore, the oral-gut microbiome axis sheds light on new clinical therapy options, and gastrointestinal tract ecology balance necessitates simultaneous consideration of both oral and gut microbiomes. This review summarizes possible routes of oral microbes entering the intestine and the effects of certain oral bacteria on intestinal microbiota and the host’s immune responses.

## 1 Introduction

For human beings, the slow-flowing and weakly acidic colon dominate the largest, densest, and most diverse microbial community, with a capacity of up to 100 billion to one trillion cells per milliliter of intestinal contents, acting as a highly efficient bioreactor (Sender et al., 2016). Functional heterogeneity of each intestinal segment determines the diversity of microbial populations in different anatomical regions, and various intestinal microbiotas sustain intestinal physiological development and epithelial homeostasis. Thus, intestinal microbiota dysbiosis is deeply correlate with gut diseases such as irritable bowel syndrome (IBS), inflammatory bowel disease (IBD), colorectal cancer (CRC), and even systemic metabolism or inflammatory diseases (e.g., rheumatism, obesity, etc.) (Priya et al., 2022). Gut microbes-derived metabolite pathways disrupt intestinal homeostasis and proceed to intestinal diseases *via* regulating the production of short-chain fatty acids (SCFAs) and trimethylamine, or distantly signal to parenteral organs and the immune system through releasing microbes or metabolites into the bloodstream (McPherson et al., 2021).

The oral and intestine are anatomically contiguous in the gastrointestinal (GI) tract, which provides chances for expanded oral pathobionts being ingested and translocated to the gut, suggesting the role of oral microbes in regulating intestinal microecology. However, many current studies separately focus on these two regions in an organ-specific manner without considering microbial translocation. The oral contains various microhabitats that are exposed or not exposed to the air. Tooth surfaces, tongue, mucous membrane, and gums are habitats of aerobe microbes whereas periodontal pockets mainly resident facultative anaerobic and anaerobic microbes, potentially act as complementary reservoirs for opportunistic enteric pathogens. However, the prerequisites for ectopic colonization of oral microbes are harsh. The majority of oral pathobionts are inactivated by gastric acidity, bile acid, colonization resistance of native microbiotas, and the immune system. As a validation, proton pump inhibitors (PPIs) users and achlorhydria patients exhibit an abnormal increase in gut colonization by oral pathobionts (Jackson et al., 2016). In recent years, experimental and sequencing research have successively revealed biological homologies between oral and intestinal flora, indicating the potential value of the oral-gut axis in precise diagnosis, effective treatment, and prognosis of gut diseases (Park et al., 2021; Bao et al., 2022). For instance, saliva could be used as a biomarker to investigate gastrointestinal status conveniently and non-invasively (Wang et al., 2021b).

Hundreds of proteins and peptides released by oral microbes could be metabolized into a variety of bioactive by-products, many of which are toxic (Barbour et al., 2022). Consequently, these metabolites possibly explain the ability that oral-to-gut microbial transmissions to shape or reshape the intestinal microbial ecosystem and eventually modulate the pathogenesis. In the intestine, oral microorganisms participate in various immunoactivity and inflammatory pathways. Saliva translocated mice intestine model shows altered cytokines, chemokines, and tight junction protein compositions, indicating the induced intestinal inflammation and enhanced mucosa permeability (Tsuzuno et al., 2021; Bao et al., 2022). The damaged intestinal mucosa increases antigen exposure, which indirectly activates immune cells and upregulates systemic inflammatory factors. In parallel, the ectopic oral pathobionts, rather than gut-resident microbes, activate the periodontist-induced Th17-skewed T cells that are imprinted with gut tropism (Kitamoto et al., 2020). Hence, regarding clinical therapy, oral microbes shall be considered synchronously and comprehensively with intestinal native microbes in designing treatment regimens.

It is not fully understood the exact oral microbes that influence gut microbiota and the mechanism of these intruders participating in intestinal diseases, further elucidations are still required. As the oral microbiome can exhibit a stable state of at least 3 months, and the gut microbiome for up to 5 years, the crosstalk between oral and intestinal microecology is pathological and clinically valuable (Faith et al., 2013; Wei et al., 2021). In this review, we highlighted the interconnections and translocation route between the oral cavity and the intestine, the immune-resistant strategy from the host, and the mechanism of the oral microorganism interacting with the intestinal ecology to provide a reference for the research frontier concerning the impact of oral microbes on intestinal microbiota.

## 2 Pathways of oral microorganisms entering the intestine

### 2.1 Enteral route

**Figure 1:**
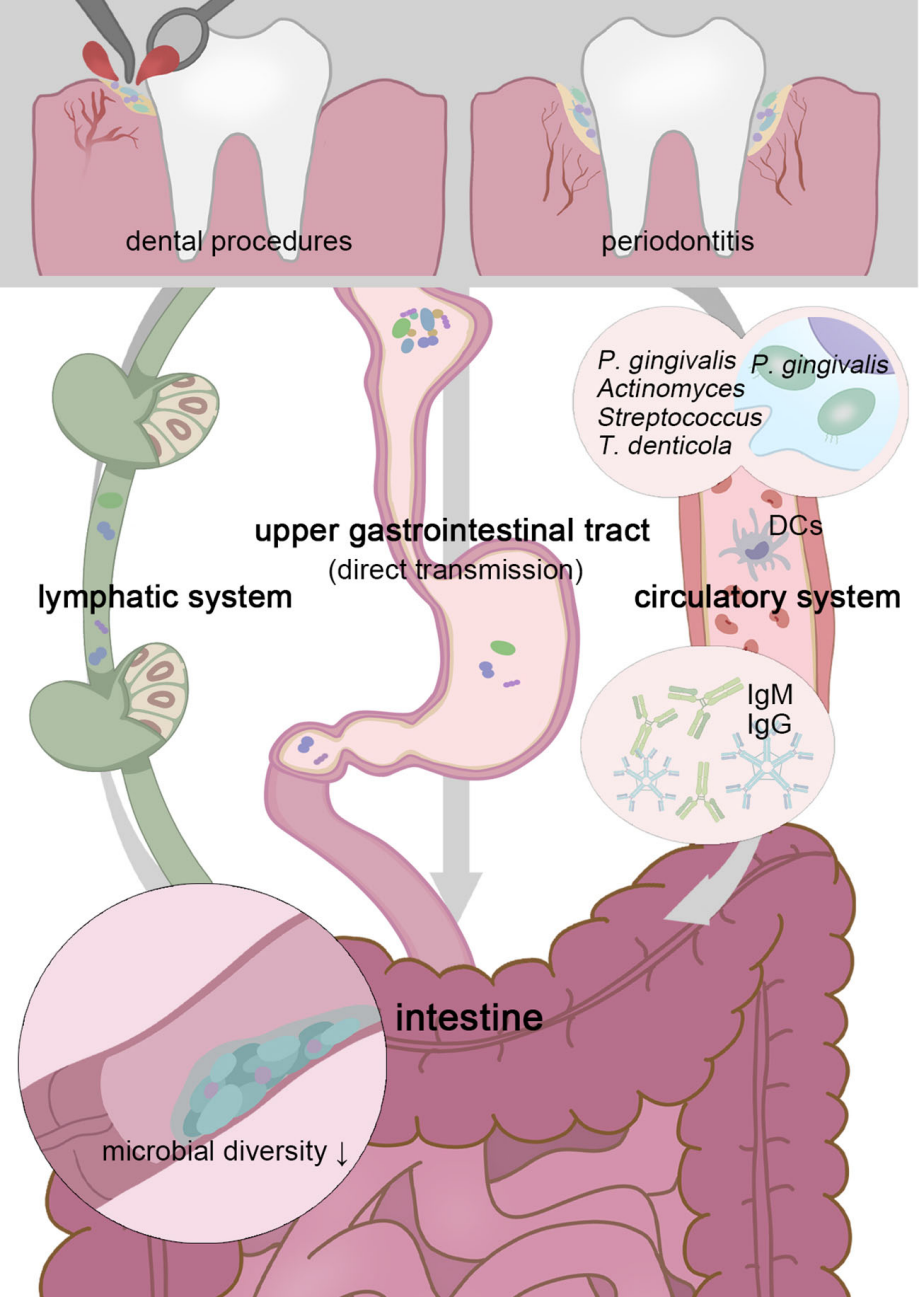
Transient physiological bacteremia triggered by periodontitis and dental procedures permits oral pathogens to stretch themselves systemically. Patients with periodontitis possess a less diverse gut microbiota, and higher possession of oral taxa in the intestine gives rise to more serious gut inflammation (Lourenvarsigmao et al., 2018). To tolerate the inactivation of stomach acid and bile acid, these unexpected “visitors” are expected to be highly acid-resistant. Moreover, *P. gingivalis* and *F. nucleatum* can be parasitic on dendritic cells or macrophages and disrupt extraoral tissues (Carrion et al., 2012; Xue et al., 2018). Oral microbes, including *P. gingivalis*, *Actinomyces*, *Streptococcus*, and *Treponema denticola*, have been confirmed as sources of non-oral organs in a colonizing manner (Figuero et al., 2014; Zhang et al., 2021; Deppe et al., 2022). Chronic oral infection establishes *F. nucleatum* colonization that significantly induces systemic humoral IgG and IgM antibody responses (Velsko et al., 2015).

Normal humans produce 0.75-1.5L saliva per day, which contains a considerable number of microbes. In mice experiments that establish oral microbial infection through oral administration or gavage of the saliva from periodontitis patients, an abnormal enrichment of oral pathobionts that were confirmed as secondary components of the salivary microbiota was detected in mice’s intestines (Atarashi et al., 2017; Bao et al., 2022). For human beings, most oral species (including core Soral taxa, *Streptococcus, Veillonella, Actinomyces*, and *Haemophilus*) that result in endogenous infection emerged in the GI tract in health and disease status, with high levels of variation across individuals (Schmidt et al., 2019) (**Figure 1**).

#### 2.1.1 Duodenum

High-throughput DNA sequencing of oral, duodenal, and pancreatic samples reveals distinct strain-level clustering patterns for each anatomical region and microbiome overlaps between them (Barlow et al., 2021). This similarity is further extended in the context of GI diseases, and absolute loads of ectopic colonized microbes are positively correlate with more severe GI symptoms. Dyspepsia patients’ duodenum components behave oral-like, including organisms from the HACEK group, *Klebsiella*, *Escherichia*, *Enterococcus*, and *Clostridium*, replace the ordinary strict anaerobes (Barlow et al., 2021; Chung et al., 2021). Moreover, automatically connected as the gate towards the pancreas, the oral bacterium in the duodenum may be capable of entering the pancreas. For instance, the periodontal microbes (*Porphyromonas gingivalis* and *Aggregatibacter actinomycetemcomitans*) have been proven positively related to pancreatic cancer risk (Fan et al., 2018; Nagata et al., 2022; Tan et al., 2022).

#### 2.1.2 Intestinal canal

The oral microflora owns the highest level of variation and the most diverse library of unique sequences, whereas this diversity gradually decreases from the oral to the distal gut, indicating that the microbiome undergoes selective pressure (Richardson et al., 2020). If successfully reaching the intestine, these intruders are usually pathogenic. In the intestine, oral-derived microbiotas create an inflammatory and immunosuppressive microenvironment suitable for tumorigenesis. It is detected the existence of oral-originated bacteria (*Fusobacterium nucleatum, P. gingivalis*, and *Parvimonas micra*) in CRC tissues, while butyrate-producing and anti-inflammatory microbes that originally lived in the intestine decreased correspondingly (Gao et al., 2021; Zhao et al., 2021). Ectopic oral microbes also participate in the pathogenic process of IBS and IBD (Liu et al., 2018; Baumgartner et al., 2021; Xia et al., 2021). Sequencing and endoscopies of IBS and ulcerative colitis (UC) patients’ intestines reveal a unique and characteristic ecological dysbiosis of dense bacterial biofilms and several biofilm-forming microbial species of dental plaques (Baumgartner et al., 2021; Xia et al., 2021). According to the sequencing of diseased full-thickness colon specimens including the neuromuscular region, the dominance of putative oral pathogens such as *Firmicutes* (*Streptococcus, Staphylococcus, Peptostreptococcus*) and *Fusobacteria* (*Fusobacterium*) potentially modulate colon motility (Dinakaran et al., 2018). Additionally, despite the shortage of research illustrating oral microbes colonizing in IBS patients’ intestines, the altered composition of oral microbes correlates with the severity of IBS (Fourie et al., 2016).

#### 2.1.3 Accessory organs of the digestive tract

The liver is one of the most-affected organs by biological network fluctuations, and the gut-liver axis is crucial in linking intestinal microbes and extraintestinal organs (Albillos et al., 2020). There are two possible clues for the involvement of oral microbes in the liver-gut axis. During periodontitis, the liver is constantly exposed to pathogenic factors from the oral, periodontitis-related systemic hypo-inflammation and chemokine potentially affect hepatic metabolic pathways. Both fecal and salivary microbiota have independent prognostic value for cirrhosis progress, specifically, *via* impaired salivary defense (Yonezawa et al., 2021; Jia et al., 2022; Saboo et al., 2022). The subgingival microbiota of cirrhotic patients consists of an uncommon bacterial community, indicating that its origin is from ecological dysregulation by immune system impairment (Jensen et al., 2018). Moreover, gut microbiota dysbiosis induced by the enteral translocation of periodontopathic bacteria weakens the mucous barrier, thus promoting the enterohepatic transfer of hepatotoxins and enterobacteria (Kuraji et al., 2021). *Via* disturbing intestinal metabolic and immune pathways, *Porphyromonas gingivalis* accelerates the progression of non-alcoholic fatty liver disease (NAFLD) (Albuquerque-Souza and Sahingur, 2022; Wang et al., 2022). Primary sclerosing cholangitis (PSC) with or without coexisting IBD is another typical manifestation of the altered gut-liver axis (Ruhlemann et al., 2019). Even in the early stages, PSC patients’ stool and mucosa significantly enrich oral pathogenic bacteria such as *Streptococcus*, *Veillonella*, and *Actinomyces* (*Streptococcus salivarius* is an independent predictor of liver-related three-year mortality) (Ruhlemann et al., 2019; Jia et al., 2022).

### 2.2 Hematogenous route

Particularly for periodontitis and periapical inflammation patients, transient physiological bacteremia triggered by tooth extraction and periodontal procedures permits oral pathogens to stretch themselves systemically *via* the circulatory system (Olsen and Yamazaki, 2019)(**Figure 1**). For severe periodontitis, the subgingival plaque has direct access to the damaged periodontal pocket, bacteria are more likely to infiltrate periodontal blood vessels and gingival epithelium in the context of the increased mucosa permeability, through which, *P. gingivalis* and *F. nucleatum* can be parasitic on dendritic cells or macrophages and disrupt extraoral tissues (Carrion et al., 2012; Xue et al., 2018). It is widely acknowledged the linkage (not causation) between periodontal disease and atherosclerotic vascular disease. Oral microbes, including *P. gingivalis*, *Actinomyces*, *Streptococcus*, and *Treponema denticola*, have been confirmed as sources of infected heart valves, atherosclerotic plaques, and rheumatic heart valves (Figuero et al., 2014; Zhang et al., 2021; Deppe et al., 2022). In this way, pathogens and their metabolites may be disseminated to the intestine similarly. Periodontal patients possess a less diverse gut microbiota, and higher possession of oral taxa in the intestine, which gives rise to more serious gut inflammation (Lourenvarsigmao et al., 2018). A retrospective analysis of individuals’ blood samples indicates that patients with *F. nucleatum* or *Streptococcus pepticus* bacteremia are more likely to suffer from subsequent colorectal cancer (Kwong et al., 2018). For clinical CRC diagnosis, plasma is more advantageous than stool in sensitivity and holds a more unique and heterogeneous microbial community, the vast majority of circulating bacterial DNA could retrospect to the gastrointestinal tract and the oral cavity (Xiao et al., 2021).

However, the accurate location of oral microbes and the probability of colonization in the distal intestine remain controversial. Precise amplicon sequence variants analysis accomplished by Armin Rashidi et al. excluded a significant distinction of microorganisms between oral and gut (66 healthy adults from two countries), indicating more clinical pathologic examinations are still required (Rashidi et al., 2021). Vasapolli et al.’ s sequencing regarding healthy subjects’ saliva, mucosa, and stool samples from each segment of the GI tract displayed an approximate absence of major species of the upper GI tract in the lower GI tract (Vasapolli et al., 2019). Similarly, the correlation between oral taxa and pancreatic cancer did not present among an African-American population as previously speculated (Petrick et al., 2022). *Via* symbiotic analysis, Carr et al. discovered contrasting ARG-species correlations between saliva and stool samples (Carr et al., 2020). Whereas most 16S rRNA gene sequencing analyses only reliably classify taxa to the genus level and do not report species and strains usually. Using shotgun metagenomic sequencing, oral and gut bacterial communities show greater similarity than earlier studies suggested (de Cena et al., 2021; Maki et al., 2021). Moreover, diurnal variations, processing methods, race, and diet are irrelevant interference with measurement results, which requires further consideration in future studies (Superdock and Poole, 2022).

## 3 Host’s immune strategy for resisting translocation

The gingiva consists of epithelium, blood vessels, lymphatic vessels, etc., which provides a physical and chemical barrier to separate the external and the internal. However, periodontal tissue is highly susceptible to dysbiosis and inflammation induced by opportunistic pathogens that reside in gingival crevicular fluid and periodontal pockets. Pathological periodontal tissues activate immune responses as the regional accumulation of leukocytes and B cells and the increase of IL-1β, IL-6, and TNF (Lu et al., 2016). Lipopolysaccharide (LPS) produced by oral microbes activates the toll-like receptors (TLR)-nuclearfactorkappa-B (NF-κB) pathway, a key factor for innate and adaptive immune responses. Moreover, it is widely acknowledged the correlation between periodontitis and systemic low-grade inflammation (e.g., Diabetes, rheumatoid arthritis, IBD, obesity, and cirrhosis) (D'Aiuto et al., 2018; Kroese et al., 2021; Moskovitz et al., 2021). During periodontitis, systemic immunity feeds back on the pathological periodontal region and affects the intestine nonspecifically. Chronic oral infection establishes ectopic oral *F. nucleatum* colonization in the intestine and significantly induces systemic humoral IgG and IgM antibody responses (Velsko et al., 2015). In mice models that are orally administrated with saliva pathogens, endotoxemia and intestinal dysbiosis are induced (Kobayashi et al., 2020). Consistent with this, periodontal therapy effectively proves the dysbiosis in stool and saliva, endotoxin, and salivary or serum inflammation factors in cirrhotics (Bajaj et al., 2018). Long-term chronic inflammation even results in an immunization training effect. Myeloid skewing of hematopoietic progenitor cell differentiation which guides the expression of various microbial product receptors, if chronically activated, potentially sustains chronicity of inflammatory pathologies (**Table 1**) (Hajishengallis and Chavakis, 2021).

**Table 1:** Experimental studies of oral microbe in intestinal ecology.

Year	Study objects	Experimental treatment	Microecology vatiations		16S rRNA Sequencing region
			Phylum	Family/Other classification methods	
2021	C57BL/6J mice	*P. gingivalis* infection		β-diversity varied	V4
2021	diabetes mice			*Prevotellaceae*↑	(metagenomic analysis)
2017	C57BL/6 mice	*P.gingivalis* infection-vs western diet		α-diversity↓	V4
2014	C57BL/6N mice	*P.gingivalis* infection (W83)-vs placebo control	*Bacteroidales*↑ (excluding *P. gingivalis*)		V5-V6
2015	C57BL/7N mice		*Bacteroidetes*↑, *Firmicutes*↓		V4
2018	C57BL/8N mice		*Deferribacteres*↑, *Bacteroidetes*↓	*Deferribacteriaceae*, *Gemellaceae*, *Clostridiaceae*↑	V4
2019	streptozotocin-induced diabetic C57BL/6J mice and wild-type mice	*P.gingivalis* infection-vs placebo control	*Deferribacteres*↑	*Lactobacillus*, *Turicibacter*↓	V1-V2
2020	C57BL/6 mice	*S. mitis, S. salivarius, P. gingivalis* or *P. nigrescen* oral infection		*Staphylococcus*, *Bacteroides*↑	V4
2022	wild-type BALB/cJ mice	Kanamycin sulfate administered and *P.gingivalis* infection		*Acholeplasmataceae*↑	V3–V4 and ITS1
2019	germ-free mice	human saliva transplantation		*Streptococcus, *Veillonella*, *Haemophilus*, *Fusobacterium*, *Trichococcus Actinomyces*↑	V4-V5
2021	C57BL/6N mice	administered either vehicle, *P. gingivalis*, or *P. intermedia*		*Eubacterium fissicatena*, *Atopostipes Colidextribacter*↑	/
2020	germ-free mice	oral pathobionts or periodontal microbe infection		*Veillonella rogosae, *Actinomyces naeslundii*	/
2022	C57BL/6J mice	intraperitoneal administration of Pg-LPS		*S24-7*↑, *Lachnospiraceae*, *Bacteroides*, *Lactobacillus*↑	V3–V4
2022	C57BL/6 mice	periodontitis individuals' saliva gavage		*Aerococcus Ruminococcus*↑	/
2022	C57BL6/J mice			*Porphyromonadaceae Fusobacterium	V4-V5
2022	C57BL/6NJcl (germ-free) mice				V4
2020	SKG mice	periodontitis induction by *P. gingivalis*	*Bacteroides*↑, *Firmicutes*, *Deferribacteres*, *Clostridiales*↓	S24-7↑	V4
2022	apoE mice			*Allobaculum*, *Akkermansia*, *Sutterella*↑	V3–V4
2020	C57BL/6 J mice	chronic periodontitis induction	*Firmicutes*, *Verrucomicrobia*, *Acidobacteria*↑, *Tenericutes*↓	*Dubosiella*, *Muribaculum*, *Butyricicoccus* ↑	V3-V4
2018	C57BL/6 J mice	endotoxemia by intravenous injection of P. gingivalis	*Tenericutes*, *Proteobacteria*↓	*Alcaligenaceae*, *Erysipelotrichaceae*↑, *Dehalobacteriaceae*, *Ruminococcaceae*↓	V3-V4
2017	CIA DBA/1J mice	*P.gingivalis* OMT	*Firmicutes*↑, *Bacteroidetes*↓	*Prevotella*, *Bacteroidetes*↓	V4
2020	induced-colitis C57BL/6 mice model	oral *Candida albicans* infection	*Bacteriodes*, *Proteobacteria*↑	*Enterobacter*, *Pseudomonas*↑	V4
2022	C57BL/6 mice	*P. gingivalis* infection-vs PBS control	*Acholeplasmataceae*	*Pyricularia pennisetigena*, *Alternaria alternata*↑	(metagenomic analysis)
2022	T1D germ-free mice	mixed saliva samples inoculation		*Klebsiella pneumoniae	V3-V4
2020	ligature-induced periodontitis model	/		*Klebsiella*, *Enterobacter*↑	V4
2021	BALB/c athymic nude mice	/		*Blautia*, *Lachnospiraceae*, *Streptococcus* (CRC locus)↑	V4
2022	APPswe/PS1ΔE9 (PAP) transgenic mice	/	*Bacteroidetes*↑, *Firmicutes*↓	*Sutterella*↑, *Lactobacillus*↓	V3-V4

(* composition variation of microbiota was detected).

**Figure 2:**
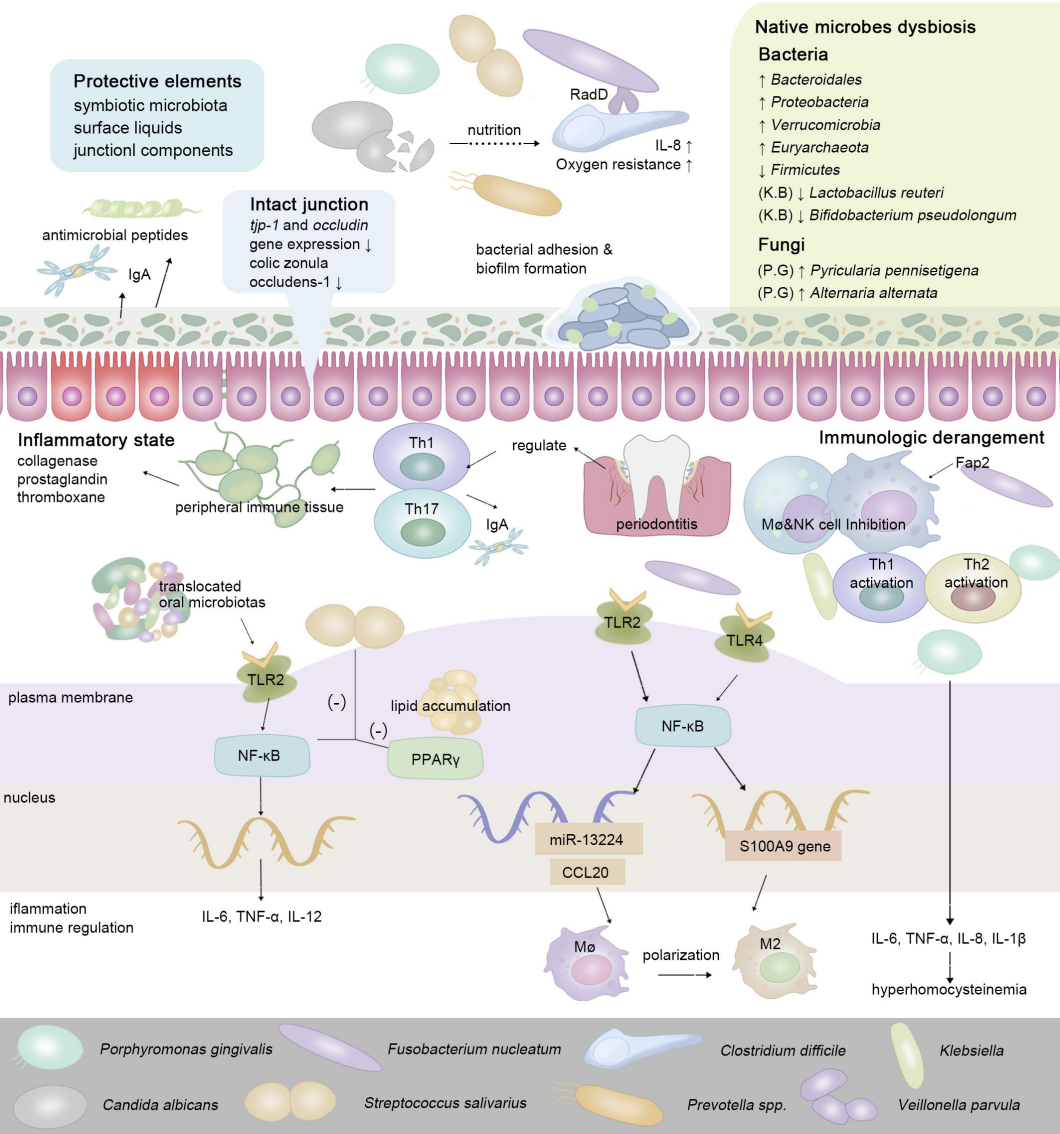
To maintain ecological balance, intestinal protective elements resist extra-intestinal microbes in the oral-derived microbiome dysbiosis. In the intestine, the enteral translocation of periodontopathic bacteria brings about gut microbiota dysbiosis and intestinal inflammation, thus weakening the intestinal surface liquids and junctional components (Nakajima et al., 2015; Feng et al., 2020). While interacting with the host’s immune system, via enteral route or hematogenous route, oral-derived pathogens regulate the constitution and transformation of immune cells, guiding the transition of the intestinal microenvironment from a healthy state to a pre-disease state. In regard to cell signaling pathways, cells of Paneth autonomously sense a series of oral metabolites via MyD88-dependent toll-like receptor (TLR), then stimulate antimicrobial cytokines and trigger the downstream NF-kB pathway, which subsequently regulates the production of a series of inflammatory cytokines (Vaishnava et al., 2008; Qi et al., 2022).

The gut has physiologically formulated multiple strategies to resist colonization by non-native bacteria. Complete mucosa protection contains the joint efforts of symbiotic microbiota, surface liquids, and junctional components (Caballero and Pamer, 2015). *Via* the gingival-intestinal axis, the swallowed saliva transmits enzymes, effector cytokines bacteria, and bioactive inflammatory cell subsets (neutrophils, lymphocytes, and macrophages) to distant locations (Byrd and Gulati, 2021). Macrophages fulfill sentinel functions as the first responder for ectopic microbes (Hausmann et al., 2021). Cells of Paneth autonomously sense LPS *via* MyD88-dependent TLR, then stimulate antimicrobial cytokines and trigger the downstream NF-κB pathway, which subsequently regulates the production of Th17 cells or Tregs (LPS from *P. gingivalis* induces a stronger Th2 response while *Klebsiella pneumoniae* induces a stronger Th1 response) (Vaishnava et al., 2008; Qi et al., 2022). Proinflammatory pathogens and metabolites gradually predispose the gut to an inflammatory state, manifesting as an enhanced expression of IL-1, IL-6, TNF-α, and vascular endothelial growth factor (VEGF), stimulating the release of collagenase, prostaglandin, and thromboxane (Akdis, 2021) (**Figure 2**). In IBD, whose major pathogenic processes involve the overreaction (hypersensitivity) of the immune response toward commensal intestinal bacteria, the mutation of the nucleotide-binding oligomerization domain 2 (NOD2) gene results in the reduction of antimicrobial peptides produced by Paneth cells (Larabi et al., 2020).

## 4 Mechanisms of oral microbiome affecting intestinal microbiota

### 4.1 Porphyromonas gingivalis

In general knowledge, *P. gingivalis* is a major pathogen for periodontitis and extensively involves in systemic diseases. To disrupt host-microbial homeostasis of the oral cavity, *P. gingivalis* only needs a microscopic colonization level (<0.01% of the total microbiota presented) (Hajishengallis et al., 2011). Extracellular translocation of *P. gingivalis*-nucleotide-diphosphate-kinase enzyme (NDKs) promotes microbial molecules to disrupt cytoplasm and establishes continuous infection in mucosal epithelial tissues (Atanasova et al., 2016). In mice experiments, a series of symptoms appeared after *P. gingivalis* infection (oral administration, gavage, or intravenous injection), including endotoxemia, intestinal inflammation, and systemic hypo-inflammation (Ohtsu et al., 2019; Kashiwagi et al., 2021). Regarding the intestine, *P. gingivalis* infection gives rise to microbiome dysbiosis. It increases serum endotoxin levels, allowing opportunistic pathogens to colonize in the oral cavity and stimulating the overgrowth of commensal microbes in the intestine (generally upregulates *Bacteroidetes* and *Deferribacteres*, and downregulates *Firmicutes*) (Nakajima et al., 2015; Ohtsu et al., 2019). *Pyricularia pennisetigena* and *Alternaria alternata* positively correlated with *P. gingivalis* administration in the fungal community. Serum metabolites are modified correspondingly with microbial alteration, especially lipid and tryptophan metabolic pathways (Chen et al., 2022). In *P. gingivalis*-administered mice, the biosynthesis of phenylalanine, tyrosine, and tryptophan in the intestinal microbiota are upregulated, with alanine, glutamine, histidine, tyrosine, and phenylalanine in serum promoted considerably in the serum (Kato et al., 2018). In terms of mechanism, *P. gingivalis* and its metabolites (e.g., gingipains and extracellular vesicles) activate antigen-presenting cells, initiating intestinal inflammation and immune reactions. *Via* stimulating TLR2/4 and inducing Th17 cells, *P. gingivalis* and its metabolites heighten the mRNA expression of TNF-α and IL-1β in the colon (Feng et al., 2020; Tang et al., 2021). Regional inflammation impairs the permeability of the intestinal barrier, specifically, downregulates *tjp-1* and *occludin* gene expression, and decreases colic zonula occludens-1 (Zo-1) level (Nakajima et al., 2015; Feng et al., 2020). Next, the altered proinflammatory cytokines mentioned above modify methionine (Met) and homocysteine (Hcy) metabolism (i.e., 1-carbon metabolism), which potentially stimulates hyperhomocysteinemia (HHcy), another mechanism participating in intestinal dysbiosis (Stanisic et al., 2021). In terms of Th17 cells, a recent study reported a noticeable synergy between periodontal tissue and gut microbes. The intestinal translocation of *P. gingivalis* enhances Th17 cell differentiation, and mature Th17 cells systemically spread to peripheral immune tissue and then exacerbate intestinal inflammation (Sato et al., 2017; Nagao et al., 2022). Consistent with this, the Th17/Treg imbalance induced by disordered intestinal microbial metabolism directly generates NAFLD in *P. gingivalis* administration mice (Yao et al., 2022). Compared to normal individuals, CRC patients’ intestines are pathologically colonized with abnormally abundant *P. gingivalis*, whose infection gives rise to a poor CRC prognosis (Gao et al., 2021). *Via* activating NLRP3 inflammatory vesicles (activated hematopoietic NOD-like receptor protein 3 inflammasomes) *in vitro* and *in vivo*, *P. gingivalis* triggers low-grade systemic inflammation and intestinal dysbiosis, promoting CRC neoplasia progression (Wang et al., 2021a). Moreover, the peptidoglycan from *P. gingivalis* molecules induces programmed cell death 1 ligand 1 (PD-L1) up-regulation in colon carcinoma cells and mediates deep inhibition of T cells (involving activation of RIP2 and MAPK signaling pathways, and NOD1 and NOD2) (Adel-Khattab et al., 2021).

### 4.2 Fusobacterium nucleatum

*F. nucleatum* normally resides in the intestine but increases synchronously with common oral microorganisms during CRC. 40% of the CRC patients detected identical *F. nucleatum* strains in both tumor tissue and saliva (Komiya et al., 2019). Moreover, successful periodontitis treatment reduces stool *F. nucleatum* levels (Yoshihara et al., 2021). Therefore, intestinal pathological *F. nucleatum* may derive from the oral. The continuum of its species and abundance in the GI tract could be an explanation. Transferring from saliva to the lower GI tract, the species diversity and abundance of *F. nucleatum* gradually dropped, indicating they resisted the selective stomach pressure (Richardson et al., 2020). Several *F. nucleatum* subspecies (*nucleatum, animalis, vincentii, polymorphum*) and potential new subspecies are isolated from the intestine of colorectal cancer patients, and the major encoding virulence factors for *F. uncleatum* showed evidence of horizontal gene transfer (Tran et al., 2022). Furthermore, intraperitoneal injection successfully established colonization of *F. nucleatum* in CRC tissue, which suggested the translocation of *F. nucleatum* through hematogenous route (Abed et al., 2020). Ponath et al. report FoxI, a conserved *F. nucleatum* oxygen-inducible picornavirus, acting as a post-transcriptional blocker of the major outer membrane porin FomA, is favorable for explicating how *F. nucleatum* colonizing in diverse segments in the intestine (Ponath et al., 2021).

In mucosal tissues, the adhered *F. nucleatum* promotes inflammation and stimulates immunoreaction. *F. nucleatum* and its outer membrane vesicles (OMVs) activate TLR/MyD88/NF-κB pathway, promoting the secretion of a series of proinflammatory cytokines, including IL-8, TNF, keratinocyte-derived chemokine (KC), IL-6, IFN-γ, and monocyte chemoattractant protein-1 (MCP-1) (Chen et al., 2020b; Engevik et al., 2021b). The colonic structure and mucus layers are damaged and infiltrated with immune cells. When regarding UC, this inflammatory process involves a molecular network including caspase activation, recruitment domain 3 (CARD3), and IL-17F (Cao et al., 2020). In addition, the TLR4-dependent mechanism (TLR4/NF-κB/S100A9 axis and NF-κB/miR-1322/CCL20 axis) promotes the M2 polarization of macrophages (M2-Mφ) and immunosuppressive effect, suggesting that oral *F. nucleatum* evolves more powerful virulence after colonization (Chen et al., 2018). Another virulence factor *Fusobacterium* produced that participates in immune regulation is the Fap2 protein, which directly interacts with TIGIT, mediates NK cell and T cell inhibition, while T-cell regulates inflammatory factors IL-10, IL-1β, and IL-6 (Gur et al., 2015; Li et al., 2019). Regarding CRC, *F. nucleatum* and its main pathogenic factors (FadA (binding E-cadherin), Fap2 (a galactose-sensitive hemagglutinin and adhesin binding TIGHT receptors), RadD (autotransporter) and FomA) recruit tumor-infiltrating immune cells, generating tumor microenvironment and participating in immunosuppression and tumorigenesis (Walencik, 2022). *F. nucleatum* modifies colonic Th17 cell frequency and IL-17 expression recombinant free fatty acid receptor 2 (FFAR2)-dependently (Brennan et al., 2021). Moreover, it positively correlated with the expression of Wnt-β-catenin (activated by FadA─E-cadherin) and REG Iα signaling, both of which effectively promotes the proliferation of sessile serrated adenoma/polyp (Rubinstein et al., 2013; Nishimura et al., 2021). *ENO1* gene and ANGPTL4 protein enhance the high glycolysis of colorectal cancer cells, and these two regulatory mechanisms could be upregulated by *F. nucleatum* either, which further support *F. nucleatum* colonization and positively collated with poor prognosis (Hong et al., 2021; Zheng et al., 2021). Besides, it is a notable phenomenon in periodontal disease that a variety of bacteria, including *F. nucleatum* and *P. gingivalis*, mutually coordinate for coexistence. Presumably, this symbiosis continues to the intestine. In the intestine, *F. nucleatum* coexists with *Clostridium* through adhesin RadD, encouraging the intestinal mucus layer’s bacterial biofilm formation in IBD patients. This symbiotic relationship accelerates the extracellular polysaccharides-producing process and chemotaxis level of *C. difficile* (Engevik et al., 2021a).

### 4.3 Klebsiella

*Klebsiella* is an opportunistic pathogen in the GI and respiratory tracts. Although a few *Klebsiella* physiologically colonizes in the intestine, in pathological states such as periodontitis, it demonstrates signs of ectopic colonization from the oral. Researchers found that saliva *K. pneumonia* of IBD patients rapidly establishes colonization in mice’s intestines, triggering a strong inflammatory response (upregulates IFN-γ, TH1 in the lamina propria, raises epithelial TNF-α mRNA expression in the proximal colon, and significantly induces IL-17, IFN-γ and CD4^+^ T cells level) (Atarashi et al., 2017). In mice models that were orally administrated with *K. pneumoniae*, the hypervirulent strain could even translocate from the intestine and lead to hepatic infections (Young et al., 2020). Th17 cell induction acts as a critical step in IBD pathogenesis and involves in regulating intestinal immunity and inflammation. Oral pathobiont-reactive Th17 cells (*Klebsiella* and *Enterobacter* species), rather than intestinal native microbes-reactive, translocate into the intestine and affect colitis progress (Kitamoto et al., 2020). Moreover, translocated *Klebsiella* promotes the secretion of IL-1β (mainly from inflammatory macrophages) *via* cysteine-11-mediated inflammatory vesicles, activating intestinal inflammasome (Kitamoto et al., 2020). For the intestine-native microbiome, in the *K. pneumoniae* infection mice model, gut microbial composition (down-regulates *Lactobacillus reuteri* and *Bifidobacterium pseudolongum*) and cecal metabolome (SCFAs) are altered (Wu et al., 2020). Whereas sufficient symbiotic microbe protection from *Bacteroidetes* could prevent the host-to-host transmission and intestinal colonization of *K. pneumoniae via* IL-36 signaling (Sequeira et al., 2020).

### 4.4 Streptococcus salivarius

Two-group pheromone-based subsystem BlpRH is the core node coordinating bacteriocin production and integrating signals of competence activation cascade, typically for *S. pneumoniae* and *S. mutans*. Whereas human co-salivary *Streptococcus* lacks functional BlpRH pairs, the couples bacteriocin production and competence commitment is directly guided by the competence signaling system ComRS, which is the undetermined basis for its optimal adaptation, potentially explaining the high prevalence of *S. salivarius* in the human GI tract (Mignolet et al., 2018). Aside from benefiting oral health, in terms of its effect on intestinal function, the *S. salivarius* in the lower gastrointestinal tract is crucial for inhabiting intestinal inflammation response. It positively adjusts microbiome composition and intestinal immunity, even in healthy individuals, the predominant intestinal *Streptococcus* species is *S. salivarius* (Kaci et al., 2014; Li et al., 2022). It is confirmed that *S. salivarius* downregulates NF-κB pathway that triggered by LPS and inhibits peroxisome proliferator-activated receptor γ (PPARγ) activation in small intestinal epithelial cells *in vitro* (Couvigny et al., 2015). Likewise, both mice experiment and metabolic gene sequencing (*I-FABP* and *Angptl4*, these gene products regulate intracellular lipid accumulation) revealed *S. salivarius’s* protective ability to diminish intestinal epithelial inflammation (Kaci et al., 2014; Couvigny et al., 2015). For non-small cell lung cancer patients, *S. salivarius* and *S. agalactiae* react upon gastrointestinal-derived CD4^+^ T cells and effectively activate monocytes to secrete higher levels of IL-6, IL-12 and TNF, or so-called, the Th1- and Th17-skewed cytokines (Ma et al., 2017). However, *S. salivarius* is also reported to disturb intestinal microbiota composition. Oral *S. salivarius* was found to be enriched and of concordant strains in both intestinal and oral microbiota in Crohn’s disease patients, and the species diversity of the infected intestine was considerably depleted (Hu et al., 2021).

### 4.5 Prevotella

Although most *Prevotella* species physiologically colonize in oral and gut mucosa, a small number of *Prevotella* show pathobiontic properties and have been confirmed to participate in opportunistic infections. Distinct from intestinal native microbes, *Prevotella* owns a unique nitrogen assimilation metabolic network that rarely limits their growth. Jong Nam Kim et al. observed different patterns of transcript abundances in genes involving ammonium assimilation between the classical “enteric paradigm” and *Pretovella* (Kim et al., 2017). *Pretovella* act as a vital keystone bacterium of the gut. Binding or attaching to other bacteria and epithelium, *Pretovella* creates a larger infection than in previously infected areas. A higher abundance of *P. intermedia* and *P. nigrescens* (*Pretovella* species that are commonly resident in the oral cavity) was observed in the intestine of CRC patients (Wirbel et al., 2019). In mice experiments, oral administration of *P. intermedia* induces NAFLD (Yamazaki et al., 2021). In terms of IBD, *Prevotella* is a potential alternative biomarker for UC, tightly matched with feces and other oral areas (Xun et al., 2018). Moreover, *Prevotella* predominates in the intestinal microbiome in the preclinical phase of rheumatoid arthritis, promoting pathological progression in a TH17 cell-dependent manner (Alpizar-Rodriguez et al., 2019; Maeda and Takeda, 2019). In a metagenome-wide association study (MWAS) of the gut microbiome from rheumatoid arthritis patients, Toshihiro Kishikawa et al. identified five species that belong to the *Prevotella* (i.e., *P. denticola, P. marshii, P. disiens, P. corporis*, and *P. amnii*) (Kishikawa et al., 2020). Additionally, the major task for intestinal *Prevotella* is metabolizing various proteins and carbohydrates. Consequently, high animal protein and saturated fat diet western people own higher diversity of inflammation-associated *Prevotella* in the oral cavity, with intestinal microbes demonstrating upregulated virulence factor and antibiotic resistance gene levels. (Prasoodanan et al., 2021).

### 4.6 Veillonella

*V. parvula* is an anaerobic commensal and opportunistic pathogen that plays a crucial role in bacteria adhesion and biofilm formation for oral and gut microbiomes. Type V secreted autotransporters that typically exist in diderm bacteria determine this character. Whereas inactivation of the gene coding for a poorly characterized metal-dependent phosphohydrolase HD domain protein in *Firmicutes* could inhibit autotransporter-mediated biofilm formation (Bechon et al., 2020). Intestinal *Veillonella* attaches to the mucosa of the distal gut and positively coexists with *Clostridioides* in *C. difficile* colonization (CDC) patients (Crobach et al., 2020). Besides, it was detected the existence of *Veillonella* and *Streptococcus* in atherosclerotic plaques, whose bacterial abundances positively correlated with their abundance in the oral cavity, indicating that oral *Veillonella* probably spread to extraoral organs *via* the circulatory system (Koren et al., 2011). *Veillonella* extensively participates in a range of diseases associated with gastrointestinal microbial dysbiosis, for instance, hepatic and gall diseases (autoimmune hepatitis, alcoholic hepatitis, biliary atresia, and PSC) (Kummen et al., 2017; Song et al., 2021). Actually, *Veillonella* is acid-sensitive, the abundance of *Veillonella* heightens when aldafermin-mediated suppressions of bile acid synthesis, particularly toxic bile acids, are inhibited (Loomba et al., 2021). When regarding IBD, the enrichment of adhesive bacteria (involving *Veillonella*) increases Th17 cell activation and luminal secretory IgA (Chen et al., 2020a; Gonzalez-Soltero et al., 2020). In periodontitis-associated type 2 diabetes (T2D), *Streptococcus* and *Veillonella* are dominant in the symbiosis network, and microbial shifts may contribute to systemic inflammation and metabolic dysfunction (Li et al., 2020; Letchumanan et al., 2022).

### 4.7 Candida albicans

It is almost impossible for fungi to survive in healthy people’s intestines, and *C. Albicans* dominate among the cultivable mycobiota that longitudinally persists. Intestinal *C. Albicans* probably as commensals constantly supplemented from the oral cavity, frequent teeth cleaning dramatically reduces fecal *C. Albicans* abundance (Auchtung et al., 2018; Raimondi et al., 2019). Whereas for immunocompromised individuals, intestinal fungi and fungal molecules could result in serious infections and produce severe bacterial sepsis *via* cytokine storm induction or macrophage-killing activity. *C. Albicans* gavage worsens the colitis in mice experiments, gut-leakage induces pro-inflammatory cytokines in the intestine and blood, which was hypothesized as a more severe translocation of LPS, serum (1→3)-β-D-glucan (BG) and bacteria from the gut into the systemic circulation (Panpetch et al., 2020). Regarding colonization and dissemination, in the gut, dead fungi are nutrients for bacterial fermentation. In the presence of *C. Albicans*, the opportunistic bacterium *C. difficile* can tolerate aerobic conditions, and the p-cresol produced by *C. difficile* inhibit *C. Albian’s* hypha formation, biofilm formation, and virulence (van Leeuwen et al., 2016). This symbiotic relationship leads to more severe infection of *C. difficile* in mice models and increases IL-8 production in experimental intestinal epithelial cell-lines (Panpetch et al., 2019). Regarding the susceptibility to *C. Albicans*, *C. Albicans* infection could be promoted by intestinal taurocholic acid (TCA) that weakens mucosal innate and adaptive immune responses (Datta et al., 2022). Besides, the host’s high iron level enhances *C. Albicans* infection severity and its dissemination from the oral to the gut (Tripathi et al., 2022). However, endogenous invasion by *C. Albicans* could be prevented by intestinal TLR2, and intravenous immunoglobulin (IVIg) has been proven useful for treating *C. Albicans* infection and maintaining intestinal homeostasis, which negatively regulates PPARγ and TLR-4 (Prieto et al., 2016; Charlet et al., 2019).

### 4.8 Other pathogens

The intestinal microecological disorder is a process affecting multi-bacterial composition. In addition to common oral pathogens mentioned above, for instance, *Peptostreptococcus stomatis* engages in gastrointestinal cancers and is of significant centralities in the gastric carcinoma (GC) ecological network, which mediates the pathogenesis of precancerous gastric atrophy (GA) and intestinal metaplasia (IM) (Coker et al., 2018; Wu et al., 2022). Regarding IBD and CRC, *P. stomatis* is among the oral-derived biomarker panel of CRC either (Yu et al., 2017; Osman et al., 2021). *Gemella* enriches in the intestinal mucosa of IBD and CRC, while *Mogibacterium* is significantly more abundant in patients with adenomatous polyps (Hale et al., 2017; Rengarajan et al., 2020; Avuthu and Guda, 2022). Intestinal bacteriophages could communicate with gut-oral bacterial commensals, which promotes individuals’ virome profile differentiation at the early and late stages of CRC (Nakatsu et al., 2018). In terms of gastrointestinal motility disorders, a polybacterial infection (*P. gingivalis*, *T. denticola*, and *Tannerella forsythia*) is discovered to alter vascular and colonic BH4/nNOS/NRF2 pathways and to damage vascular relaxation and colonic motility (Gangula et al., 2015). In addition, oral lantibiotics (it has a considerable antimicrobial effect on intestinal gram-positive bacteria) produced by *S. mutans* decrease the diversity of intestinal microbes and reduce the abundance of *Firmicutes* (Yonezawa et al., 2021).

## 5 Conclusion and perspectives

Growing evidence supports the associations between oral microbes and intestinal diseases. The prevalence and severity of oral diseases (e.g., periodontitis) are closely linked with the intestinal healthy state. In recent years, several intestinal pathobionts (*P. gingivalis*, *F. nucleatum*, *P. intermedia*, *P. nigrescens*, *C. albicans*, etc.) showed potential origin from the oral cavity *via* direct enteral translocation or through the circulatory system. Using deep learning algorithms, the fecal microbiome could be predicted by the oral microbiome of the same individual in a new bioinformatic tool (Rampelli et al., 2021). For enteral ectopic translocation, oral microbes have to overcome the harsh protective elements (e.g., gastric acid, bile acid, and native microbes). Consequently, the detected oral microorganisms in the lower GI tract are usually low-abundant. With the development of next-generation sequencing (NGS), the consistency between the two sites is more detectable and demonstrates further correlations than in earlier studies. In mice experiments, oral administration of oral pathobionts leads to intestinal dysbiosis, regional intestinal inflammation, and low-grade endotoxemia. Although the amount and method of intake differ from the natural state, it provides references to the possible effect of oral pathobionts on the gut, and more experiments are required to determine the species and proportion of pathobionts, the precise site of their colonization, the time of their presence, and the crosstalk with native microbes and the immune system. The circulatory system, as a channel for bacterial expansion, also facilitates the widespread dissemination of oral pathobionts. Periodontitis is generally accepted associating with systemic low-grade inflammatory diseases. The footprint of the oral-origin microbiome has been detected in extraoral organs including the heart and aortic valves. Oral microbes and their products induce immune responses *via* the bloodstream, which regulates the host’s overall inflammatory state, stimulating TLR-NF-κB pathway or regulating TH17/Treg ratio. In addition, it is a notable phenomenon for pathogenic microbes such as *F. nucleatum*, *C. difficile*, and *P. gingivalis* communicating and adhering with each other. In mucosal biofilms, interspecies interactions that influence spatial organization modify microbial communities’ formation and function. Hence, it is also interesting to investigate how these oral microbes influence the survival of native microorganisms.

16S rRNA sequencing and conventional bacterial identification and quantification methods are suitable for identifying well-characterized and abundant genera. However, frequently using 1% as the threshold for excluding the low-abundant organisms, some oral-origin microbiome species in the intestine may escape detection unconsciously. The SourceTracker results showed that the percentage of intestinal bacteria from the tongue-coated microflora was within 1% (Guo et al., 2022). Moreover, diet and diurnal variation are independent variables that affect the composition of microbes in the GI tract, which needs further consideration while analyzing microbial communities.

The study of oral-gut microbes has gained popularity, not only for its role in intestinal and systemic diseases that have gradually been explored but also for its offering new approaches to conveniently detectable biomarkers and clinical therapy methods. Various probiotics, prebiotics, and even herbal medicines that work by modifying gut microbes’ variations have been proven of positive therapeutic outcomes. Both saliva and gut microbes are potential biomarkers for diagnosis. This clue indicates an overall idea to manage oral health and intestinal diseases coordinatingly in the future, and this approach is still in its infancy. As the attention to oral health grows, studying the effect of oral microbes on the intestine opens up new insight into preventing and controlling intestinal diseases and discovering new therapies.

## Author contributions

YL, ZL, and XP wrote and edited the manuscript. ZL and XP critically reviewed the manuscript and provided insightful discussions and ideas. YL and ZL contributed equally to this work and share first authorship. All authors contributed to the article and approved the submitted version.
